# Molecular surveillance of *Helicobacter* species with high prevalence from two streams with various wastewater pollution in Taiwan

**DOI:** 10.1016/j.onehlt.2024.100757

**Published:** 2024-05-16

**Authors:** Xuan-Di Cao, Ya-Ling Huang, Jung-Sheng Chen, Chien-Sen Liao

**Affiliations:** a Institute of Biotechnology and Chemical Engineering, I-Shou University, Kaohsiung 840203, Taiwan; b Department of Laboratory Medicine, E-Da Hospital, I-Shou University, Kaohsiung 824005, Taiwan; c Department of Medical Laboratory Science, I-Shou University, Kaohsiung 824005, Taiwan; d Department of Medical Research, E-Da Hospital, I-Shou University, Kaohsiung 824005, Taiwan; e Department of Medical Science & Biotechnology, I-Shou University, Kaohsiung 824005, Taiwan; f Institute of Biopharmaceutical Sciences, National Sun Yat-sen University, Kaohsiung 804201, Taiwan

**Keywords:** *Helicobacter* species, Gastric *Helicobacter*, Enterohepatic *Helicobacter*, Stream water, Livestock sewage

## Abstract

*Helicobacter* species are potential zoonotic pathogens classified as either enterohepatic or gastric. *Helicobacter* infection can be transmitted through wastewater from households and livestock and through water from irrigation and streams. In this study, the distribution and source of *Helicobacter* species in the Donggang and Yenshui rivers, two natural water bodies with different characteristics, were analyzed. A total of 44 water samples were collected over the four seasons. The samples were subjected to *Helicobacter* 16 s rRNA gene PCR, followed by sequencing and comparison for identification and analysis. The detection rate of *Helicobacter* species in both rivers was 79.55%, with *H. kayseriensis* (10/35, 28.57%) being the most common species. Analysis of the environment around the sampling sites showed a high detection rate in the livestock-rich area, and the results of BLAST for species identification and comparison indicated feces as the contamination source. The area around the Donggang River was developed for animal husbandry, led to a high detection rate of *Helicobacter* species. Many *Helicobacter* species were identified to have a risk of zoonotic transmission, especially if the stream is used as a source of drinking, agricultural, or even aquacultural water. The high presence of *Helicobacter* species in natural water bodies suggests that wastewater treatment is an effective strategy to control pathogen spread. Therefore, investigation and monitoring of pathogens in wastewater are highly important. However, methods for the isolation and culture of *Helicobacter* species in natural waters have yet to be developed. Hence, future research should focus on developing such methods.

## Highlights

• Only amplicon TKC5-Sp belonged to the gastric *Helicobacter* group.
• *Helicobacter kayseriensis* was the most prevalent species in the two streams.
• *Helicobacter* species were detected in the Donggang River, a drinking water source.
• Significant difference was found between pH and *Helicobacter* presence.

## 1 Introduction

*Helicobacters* is a genus of gram-negative spiral bacteria that are mainly found in the stomach and intestines of various animals [1,2]. Species of this genus can be classified as either gastric or enterohepatic. *Helicobacter pylori* is responsible for many gastric diseases, such as chronic gastritis and gastric ulcer, which may lead to gastric cancer [3]. Among gastric *Helicobacter* species, non—*H. pylori Helicobacter* (NHPH) species, including *H. heilmannii*, *H. felis*, and *H. suis*, have attracted considerable attention. *H. suis* is the most common NHPH in the human stomach, indicating that it possesses the risk of zoonotic transmission and predisposes individuals to gastritis, gastric ulcers, and mucosa-assisted lymphoid tissue [4]. Enterohepatic *Helicobacter* species include *H. bilis*, *H. canis*, and *H. winghamensis*. Among them, *H. winghamensis* is widely found in wild rodent species. It has also been identified in the feces of patients with gastroenteritis, indicating that it may serve as an indicator of gastrointestinal inflammatory diseases and possess zoonotic properties [5]. Enterohepatic *Helicobacter* species may be one of the most significant pathogens of inflammatory bowel disease [6].

The infection rate of gastric *H. pylori* is unevenly distributed worldwide, e.g., 30% in the United States, 44.2% in mainland China, 45.1% in Taiwan, and 40.6% in Korea [[7], [8], [9]]. Moreover, infection rates may differ within the same region, suggesting that the conditions of *H. pylori* infection are closely related to environmental variations and host genetics [10]. Urea breath test results revealed a 45.1% prevalence rate for *H. pylori* infection in Taiwan in 2020 [7,8]. In the same country, drug-resistant *H. pylori* infections have also been identified [11]. Based on the zoonotic tendency of *H. pylori*, the high similarity of *Helicobacter* species in canines and human owners in Taiwan suggests cross-transmission between canines and humans [2]. Meanwhile, the high prevalence of *H. pylori* infection in southern Uganda may be due to a lack of clean water, poor sanitation, and overcrowding of the population [12]. This finding suggests that the pathogen may spread through water, infecting both animals and humans. Therefore, a comprehensive understanding of the transmission routes, types, and modes of *Helicobacter* infections can contribute to improving One Health approaches and developing effective preventive strategies.

Although the transmission routes of gastric *H. pylori* and other *Helicobacter* species have not been conclusively established, the main transmission routes include human–human, animal–human, food, and water [13,14]. Genus *Helicobacter* is also found in various aquatic environments, including rivers, seawater, and irrigation water [15,16]. Food products, such as milk, fresh vegetables, and meat, also play an important role in the transmission of *Helicobacter* species [17]. Aquatic environmental factors may also be responsible for the wide distribution of *Helicobacter* species. Such diverse routes of *Helicobacter* transmission pose significant challenges to achieving the goal of environmental One Health.

Streams and rivers are the confluence of many sources of wastewater or natural water and are often used as a source of aquaculture, irrigation, or even drinking water. The cleanliness and water quality of streams and rivers are important for controlling human diseases. Stream pollution can be determined by microbial species, and the surrounding environment can provide an overall status of stream pollution. Considering that *Helicobacter* infections are potentially zoonotic, we focused our investigation on two watersheds, the Yanshui and Donggang rivers, located in Southern Taiwan. These watersheds are surrounded by farms and residential areas, which are sources of wastewater. Both areas have sources of water for domestic use, irrigation, and aquaculture.

Therefore, the main objective of this study is to analyze the distribution and types of *Helicobacter* species in both streams to gain insights into the possible sources of wastewater and pathways of pathogen transmission. The results of this study will serve as a basis for developing strategies to manage and control the contamination of pathogens, specifically *Helicobacter* species, and promote One Health.

## 2 Materials and methods

### 2.1 Water sampling

**Fig. 1:**
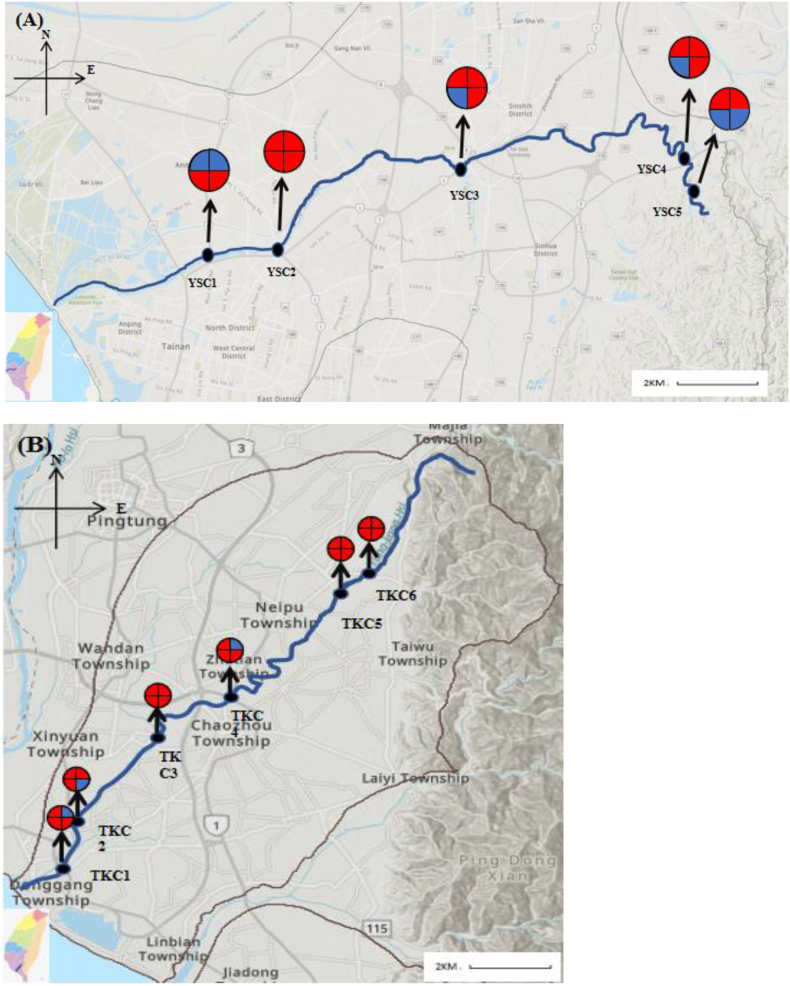
Distribution of *Helicobacter* in the Yan-Shui Creek(A) and Dong-gang Creek(B). Note:  Pie chart denotes the seasonal occurrence of *Helicobacter*; 1- Spring; 2- Summer; 3-Autumn; 4- Winter; *Helicobacter* positive; *Helicobacter* negative.

In this study, water samples from Donggang River in Pingtung City and Yanshui River in Tainan City were collected over four seasons and analyzed (Fig. 1). Sampling locations were selected by investigating the watershed distribution of the Yanshui and Donggang rivers, the locations of the trails, and the environmental and residential conditions in the areas through which these two rivers pass. Donggang River spans a length of 44 km, with a drainage area of 472.20 km^2^, and its geographic coordinates range from (120.4367, 22.4677) to (120.6431, 22.6261). The main sampling points in this study, from upstream to downstream, were the Chengde Bridge (TKC6), Longdong Bridge (TKC5), Chauchou Bridge (TKC4), Xingshe Bridge (TKC3), Gangxi Pumping Station (TKC2), and Donggang Bridge (TKC1). TKC6 and TKC5 are in the upper reaches of the river and are surrounded by farmland and livestock farms, whereas TKC4 and TKC3 are in the middle reaches of Donggang River and are surrounded by a large number of farms and large-scale livestock farms (Data from Animal Genetic Resources Information Network in Taiwan). In the downstream area, TKC2 and TKC1 are surrounded by a large number of residential areas, and a well-developed domestic sewage system can be found on the east bank of TKC1 but not on the west bank (Data from Construction and Planning Agency Ministry of The Interior). Yanshui River spans a length of 41.30 km, with a drainage area of 339.74 km^2^, and its geographic coordinates range from (120.1398, 22.9977) to (120.3718, 22.9804). The main sampling points from upstream to downstream in this study were Tongxin Bridge (YSC5), Qianniao Bridge (YSC4), Fenghua Bridge (YSC3), Xidingliao Bridge (YSC2), and Yanshui river Bridge (YSC1). YSC5 and YSC4 are in the upper reaches of the Yanshui River, with a small number of farms and livestock farms around them, whereas YSC3 and YSC2 are in the middle reaches of the Yanshui River, with a large number of farms and livestock farms around them (Data from Animal Genetic Resources Information Network in Taiwan). YSC1 is in the lower reaches; the southern part of the river is densely populated and has a comprehensive domestic sewage system, whereas the northern part of the river has aquaculture farms (Data from Construction and Planning Agency Ministry of The Interior, Taiwan).

### 2.2 *Helicobacter* and *Helicobacter pylori* PCR

In this study, *Helicobacter* species were detected using 16 s rRNA as previously described [18]. PCR was performed using a modified forward primer C97b-F: GCTATGACGGGGTATCCGGC and the existing reverse primer C05-R: ACTTCACCCCAGTCGCTG. Amplification conditions included denaturation at 94 °C for 3 min, followed by 35 cycles of denaturation at 94 °C for 30 s, annealing at 58 °C for 30 s, an extension of 72 °C for 60 s, and a final extension of 72 °C for 10 min. This procedure yielded a 1200 bp PCR amplification product. Nested PCR was performed using the modified forward primer C97b-F: GCTATGACGGGGTATCCGGC and the existing reverse primer C98-R: TGGTGTAGGGGGTAAAATC. Amplification conditions included denaturation at 94 °C for 3 min, followed by 35 cycles of denaturation at 94 °C for 30 s, annealing at 55 °C for 30 s, extension at 72 °C for 60 s, and a final extension at 72 °C for 10 min. This procedure yielded a 389 bp PCR amplification product. The *ureC* and *vacA* genes were detected as previously described [19]. PCR was performed using a modified forward primer *ureC*-F: TTATCGGTAAAGACACCAGAAA and the existing reverse primer *ureC*-R: ATCACAGCGCATGTCTTC. Amplification conditions included denaturation at 95 °C for 10 min, followed by 40 cycles of denaturation at 94 °C for 10 s, annealing at 54 °C for 5 s, an extension of 72 °C for 8 s, product detection at 77 °C for 5 s, and a last cooling step at 4 °C for 60 s. This yielded a 132 bp PCR amplification product. PCR was performed using a modified forward primer *vacA*-F: ATGGAAATACAACAAACACAC and the existing reverse primer *vacA*-R:CTGCTTGAATGCGCCAAAC. Amplification conditions included denaturation at 95 °C for 10 min, followed by 35 cycles of denaturation at 94 °C for 30 s, annealing at 51 °C for 60 s, extension at 72 °C for 60 s, and a final extension at 72 °C for 10 min. This yielded a 259 bp PCR amplification product.

### 2.3 Sequencing and phylogenetic analysis

The 16 s rRNA PCR products were sequenced using genomics. The sequences obtained were submitted to NCBI GenBank and then used to identify *Helicobacter* species using MEGA11 for subsequent phylogenetic analysis. After matching the characteristic sequences from NCBI GenBank, the sequences were compared using Muscle Alignment. Finally, a neighbor-joining algorithm with 1000 bootstrap replicates was used to construct a phylogenetic tree.

### 2.4 Statistical analysis

Mann–Whitney non-parametric test was performed using XLSTAT software (Data Analysis and Statistical Solution for Microsoft Excel, Addinsoft, Paris, France 2017) for compare differences between the presence/absence of *Helicobacter* species and water quality parameters. Statistical significance was set at *p* < 0.05.

## 3 Results

### 3.1 Prevalence of *Helicobacter* amplicons

In this study, 44 water samples were collected, and nested PCR sequencing was performed based on the 16 s rRNA gene of *Helicobacter*. *H. pylori* was not detected by the presence of *ureC* or *vacA*. Of these 44 samples, 35 were PCR positive, and the prevalence of *Helicobacter* species was 79.55% (Table 1). The results of the one-year seasonal survey showed that the overall detection rate was higher in Donggang River (87.5%) than in Yanshui River (70%). Based on the seasonal distribution, except for the case of the Yanshui River watershed in the fall, where the detection rate was lower than 50%, the detection rates in all other seasons exceeded 66.7%. In general, the levels of wastewater pollution with *Helicobacter* species could be higher in Donggang River than in Yanshui River. Therefore, the sources of wastewater pollution in the vicinity of different possible sampling sites were investigated, and the results are shown in Table 2.

**Table 1:** Seasonal detection rates of *Helicobacter* spp. in the YanShui river and Donggang river.

Area	Season	Text positive No.	Total	Detection rate
Yan-Shui Creek (*n*^a^ = 20)	Spring	4	5	80%
	Summer	4	5	80%
	Autumn	2	5	40%
	Winter	4	5	80%
	Total	14	20	70%
Dong-gang Creek (*n*^a^ = 24)	Spring	6	6	100%
	Summer	4	6	66.67%
	Autumn	6	6	100%
	Winter	5	6	83.33%
	Total	21	24	87.5%
	Total	35	44	79.55%

a The total collection sample amount is combined from each season and the collection is the same over each season.

**Table 2:** The detection rate of *Helicobacters* across the different Potential source of wastewaters from two rivers.

	Wastewater source	Sample in site	Text positive No.	Detection rate
Potential source of wastewaters	Agricultural and Livestock pollution	TKC6,TKC5,TKC4,TKC3, YSC5,YSC4,YSC3,YSC2	28	63.64%
	Aquaculture pollution	YSC1	4	9.1%
	Domestic sewage pollution	TKC1,TKC2,YSC1	8	18.2%

### 3.2 Impact of water quality indicators on *Helicobacter* species

The water quality index and the presence or absence of *Helicobacter* species were analyzed using Mann–Whitney non-parametric analysis, and the results are shown in Table 3. Variables, including total coliform count, dissolved oxygen, conductivity, temperature, water temperature, and river pollution index (*p* = 0.424, 0.612, 0.987, 0.443, 0.100, 0.087, respectively) were analyzed in this study. 0.100, *p* = 0.087) were not significantly associated with the absence or presence of *Helicobacter* amplicons, and only pH (*p* = 0.047) was significantly associated with the absence or presence of *Helicobacter* amplicons. However, the pH values in samples, whether *Helicobacter* amplicons are present or absent, consistently fall within the range of pH 7–8, with similar median values. Thus, despite significant differences in pH, there appears to be no clear preference for acidic or alkaline environments for *Helicobacter* spp.

**Table 3:** BLAST comparisons of the creek strains from the Donggang River and YanShui River.

Strain (accession no.)	Top BLAST hit^a^	Max identity (%)	Natural host	Reference country	Reference source
TKC6-Sp	*Helicobacter trogontum* AY686609.1	398/398(100%)	Rat intestine	USA	Dewhirst F E et al. (1996)
TKC5-Sp	*Helicobacter turcicus* NR_181839.1	394/400(99%)	Anatolian Ground Squirrel	USA	Aydin,F., et al. (2001)
TKC4-Sp	*Helicobacter valdiviensis* KX503247.1	394/398(99%)	Feces of humans	Thailand	Wangroongsarb P,et al. (2017)
TKC3-Sp	*Helicobacter kayseriensis* NR_181912.1	398/398(100%)	*Pica pica*	USA	Aydin F, et al. (2022)
TKC2-Sp	*Helicobacter kayseriensis* NR_181912.1	397/398(100%)	*Pica pica*	USA	Aydin F, et al. (2022)
TKC1-Sp	*Helicobacter rodentium* AY631957.1	393/398(100%)	Rat intestine	USA	Dewhirst F E, et al.(2005)
YSC5-Sp	*Helicobacter valdiviensis* KX503247.1	398/398(100%)	Feces of humans	Thailand	Wangroongsarb P, et al. (2017)
YSC4-Sp	*Helicobacter valdiviensis* KX503247.1	396/398(99%)	Feces of humans	Thailand	Wangroongsarb P, et al. (2017)
YSC3-Sp	*Helicobacter anseris* NR_043798.1	398/398(100%)	*Branta candensis*	Canada	Fox JG., et al. (2006)
YSC2-Sp	*Helicobacter rodentium* AY631957.1	391/398(98%)	Rat intestine	USA	Dewhirst F E, et al.(2005)
YSC5-S	*Helicobacter* sp. KJ081209.1	391/398(98%)	*Tupinambis merianae*	Netherland	Gilbert, M. J, et al.(2014)
YSC4-S	*Helicobacter* sp. GU902718.1	388/398(97%)	Prairie dog	USA	Beisele,M., et al.(2011)
YSC3-S	*Helicobacter rappini* AY034819.1	394/398(99%)	Finnish porcine	Finland	Hanninen,M.L. et al.(2003)
YSC2-S	*Helicobacter rodentium* AY631957.1	393/398(99%)	Rat intestine	USA	Dewhirst F E, et al.(2005)
TKC6-S	Uncultured bacterium AY592572.1	352/395(89%)	N/A^b^	N/A^d^	N/A^b^
TKC5-S	Uncultured bacterium AY592572.1	355/394(90%)	N/A^b^	N/A^d^	N/A^b^
TKC3-S	*Helicobacter* sp. KJ081208.1	396/397(99%)	*Cordylus warreni*	USA	Gilbert, M.J., et al.(2014)
TKC2-S	*Helicobacter* sp. KJ081208.1	396/397(99%)	*Cordylus warreni*	USA	Gilbert, M.J., et al.(2014)
TKC6-A	*Helicobacter kayseriensis* NR_181912.1	396/398(99%)	*Pica pica*	USA	Aydin F et al. (2022)
TKC5-A	*Helicobacter rodentium* AY631957.1	395/400(99%)	Rat intestine	USA	Dewhirst F E, et al.(2005)
TKC4-A	*Qipengyuania citrea* CP098494.1	379/417(91%)	N/A^b^	N/A^b^	N/A^b^
TKC3-A	*Helicobacter kayseriensis* NR_181912.1	398/398(100%)	*Pica pica*	USA	Aydin F, et al. (2022)
TKC2-A	*Helicobacter kayseriensis* NR_181912.1	398/398(100%)	*Pica pica*	USA	Aydin F,et al. (2022)
TKC1-A	*Helicobacter kayseriensis* NR_181912.1	397/398(99%)	*Pica pica*	USA	Aydin F, et al. (2022)
YSC1-A	Uncultured bacterium AY592572.1	351/394(89%)	N/A^b^	N/A^b^	N/A^b^
YSC2-A	Uncultured bacterium AY592572.1	352/394(89%)	N/A^b^	N/A^b^	N/A^b^
YSC1-W	*Helicobacter kayseriensis* NR_181912.1	398/398(100%)	*Pica pica*	USA	Aydin F et al. (2022)
YSC2-W	*Qipengyuania citrea* CP098494.1	373/419(89%)	N/A^b^	N/A^b^	N/A^b^
YSC4-W	*Helicobacter rappini* AY034821.1	390/398(98%)	Finnish porcine	Finland	Hanninen,M.L., et al.(2003)
YSC3-W	*Helicobacter trogontum* AY686609.1	398/398(100%)	Rat intestine	USA	Dewhirst F E et al. (1996)
TKC6-W	*Helicobacter kayseriensis* NR_181912.1	397/398(99%)	*Pica pica*	USA	Aydin F, et al. (2022)
TKC5-W	*Helicobacter kayseriensis* NR_181912.1	397/398(99%)	*Pica pica*	USA	Aydin F, et al. (2022)
TKC4-W	*Uncultured bacterium* AY592572.1	352/394(89%)	N/A^b^	N/A^b^	N/A^b^
TKC3-W	*Helicobacter kayseriensis* NR_1819120.1	397/398(99%)	*Pica pica*	USA	Aydin F,et al. (2022)
TKC1-W	*Qipengyuania citrea* CP098494.1	373/419(89%)	N/A^b^	N/A^b^	N/A^b^

a Top BLAST hit in second column indicates the closest reference species to the environmental strain by the BLAST search. Max identity represents the percentage of maximum identity between the environmental strain and closest reference species. The fourth and fifth columns show the Natural host and country source of the closest referencespecies, respectively.

b N/A show the bacteria cannont find the Natural host and country source of the closest referencespecies, respectively.

### 3.3 Species identification and phylogenetic analysis using 16S rRNA sequencing

All 35 PCR-positive amplicons were sequenced and analyzed by BLAST using the NCBI database, and the sequence similarity ranged from 89% to 100%. As shown in Table 4, 27 sequences had a similarity of 97% or more, and 10 sequences had a maximum similarity of 100%. Among these 35 sequences, 8 amplicons were the most similar to non-*Helicobacter* species, namely, 5 strains of uncultured bacteria and 3 strains of *Qipengyuania citrea*, but the similarity (homology) was relatively low (i.e., only 89%–91%). One of the 27 amplicons was classified into the gastric *Helicobacter* group, with *H. turcicus* as the major type, and the 26 remaining were classified into the enterohepatic *Helicobacter* group (26/35, 74.29%), which included *H. valdiviensis*, *H. kayseriensis*, *H. rodentium*, *Helicobacter* sp., *H. rappini*, *H. anseris*, and *H. trogontum* (Table 3). *H. kayseriensis* (10/35, 28.57%) was the most common *Helicobacter* species in this study. It was most commonly isolated from pigs (12/35, 34.28%), followed by rat intestine (6/35, 17.14%), and human feces (3/35, 8.57%). However, the main source countries with the highest degree of sequence matching were the USA and Finland.

**Table 4:** Nonparametric statistical analysis of the presence and absence of the *Helicobacters* in relation to seven water quality parameters.

Water quality indicators	Mann-Whitney *U* test	Helicobacter-Negativity sample(*n* = 9)				Helicobacter-Positivity sample(*n* = 35)			
		Range	Median	Q1	Q3	Range	Median	Q1	Q3
Total coliform(CFU/100 mL)	p = 0.424	1300.000–600,000.000	32,000.000	5900.000	77,000.000	520.000–1,600,000.000	37,000.000	10,000.000	120,000.000
Dissolved oxygen(mg/L)	*p* = 0.612	3.000–8.800	6.400	4.100	7.400	1.700–9.000	4.800	3.600	5.900
Conductivity(μmho/cm25°C)	*p* = 0.987	370.000–25,300.000	582.500	541.250	4237.500	266.000–36,100.000	603.000	421.000	2330.000
pH	p = 0.047⁎	7.480-8.360	7.680	7.620	8.010	7.090–8.380	7.570	7.390	7.780
Temperature(°C)	*p* = 0.443	26.500–36.000	31.000	28.950	33.900	22.900–36.600	30.600	28.600	32.400
Water temperature(°C)	*p* = 0.100	25.800–32.400	30.200	27.400	31.100	24.500–32.700	28.600	27.000	29.500
River Pollution Index	p = 0.087	1.000–5.000	3.750	1.000	5.000	1.000–7.940	4.250	2.750	5.500

⁎ p < 0.05.

**Fig. 2:**
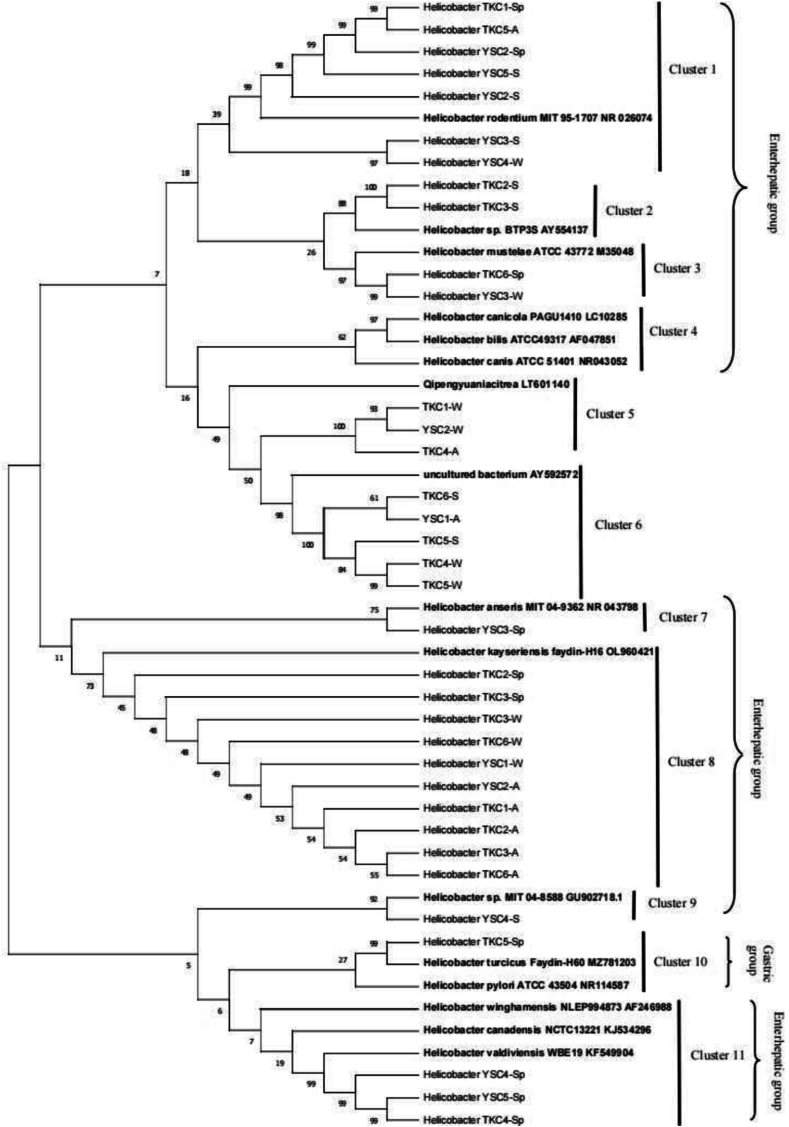
Phylogenic analysis of the *Helicobacters.* The phylogenic tree was established based on the 389 bp 16S-rRNA gene sequences. Bootstrap values (%) using 500 pseudoreplicates for the major lineage are indicated at each branch nodes. Cluster 1，2，3，4，7，8，9 and 11(Enterohepatic *Helicobacter group*)consists of *Helicobacters* from area Yan-Shui Creek and Dong-gang Creek. Cluster 10(Gastric Helicobacter *group*) consists of *Helicobacters* from area Dong-gang Creek and a USA reference strain associated Anatolian Ground Squirrel from GenBank. Evolutionary analyses were conducted using MEGA11.The reference strains were downloaded from GenBank with their accession numbers.

A phylogenetic tree was constructed using 16 s rRNA for 35 amplicons combined with sequences reported in the literature using MAGE11 to determine the molecular and evolutionary relationships among the *Helicobacter* species, which were divided into 10 clusters. As shown in Fig. 2, Clusters 1–3 and 7–11 contained one reference sequence for *Helicobacter*, Cluster 6 contained the reference sequence for an uncultured bacterium with accession number, AY592572.1 [20], and Cluster 5 contained the reference sequence for *Q. citrea* with accession number [21]. Clusters 1, 2, 3, 4, 7, 8, 9, and 11 were categorized into the enterohepatic *Helicobacter* group (26/35, 74.29%) and Cluster 10 into the gastric *Helicobacter* group (1/35, 2.86%). Cluster 8 was the most commonly detected *Helicobacter* (10/35, 28.57%). This cluster had high similarity to *H. kayseriensis* faydin-H16. Cluster 1 was the second most frequently detected *Helicobacter* species (7/35, 20%), and it had high similarity to *H. rodentium*.

## 4 Discussion

In this study, *Helicobacter* species were frequently found in both rivers, making these pathogens among the most threatening zoonotic pathogens under the concept of One Health [22]. *Helicobacter* species are present in most animals, such as dogs, cats, poultry, and even birds [2]. In fact, one study tested 80% of poultry positive for *Helicobacter* species [23], suggesting a high prevalence of this group of pathogens in different environments, including groundwater [24], soil [25], and common freshwater [26]. Pérez et al. [25] detected *Helicobacter* species in domestic soil from Spain (9/78, 11.54%), where the soil was sampled from an area where household pets were active, suggesting that pet feces might have contributed to the soil environment being infected with *Helicobacter* species. Meanwhile, in Japan, plankton and other types of bacteria present in deep groundwater and seawater environments can sustain the proliferation of *Helicobacter* species for long periods of time [24], making cross-transmission between animals and the environment a feature of concern. Moreover, *Helicobacter* species have been found present in a drinking water treatment plant in Colombia and subsequently detected using fluorescence in situ hybridization in the inlet (35/155, 22.6%) and outlet (42/155, 27.1%) of a water treatment plant [27]. This study suggests that wastewater is an important vehicle for the transmission of *Helicobacter* species and that conventional wastewater treatment systems are insufficient to eradicate *Helicobacter* species in wastewater. Surprisingly, *Helicobacter* species have also been detected in bottled drinking water samples (8/450, 1.77%), and their abundance peaks occur in July, with a 50% positive rate [28]. This result can be attributed to the temperature in July being favorable for the proliferation of *Helicobacter* species and the substandard quality of bottled water and lack of effective disinfection methods.

Considering that animal feces are an important medium for the transmission of *Helicobacter* species, we attributed the high detection rate (79.55%) of *Helicobacter* species in the Yanshui and Donggang Rivers to the surrounding livestock farming areas. This high detection rate is a serious issue that warrants continuous monitoring, especially since the Yanshui River has been marked as a future source of potable water and a portion of the Donggang River has been used as a source of tap water. In addition, these water bodies are used as sources of water for aquaculture, and important fishing ports in Taiwan are located downstream of the Donggang River. Therefore, *Helicobacter* pathogens are likely to affect the human body through aquaculture, especially through the consumption of aquatic products, such as shellfish, which are filter-feeding organisms, posing a significant environmental health problem.

In the present study, total coliform count, dissolved oxygen, conductivity, pH, temperature, water temperature, and river pollution index were measured to determine whether they are important factors influencing the presence of *Helicobacter* species in a watershed. Overall, only pH affected (*p* < 0.05) the presence of *Helicobacter* species in the watersheds. However, the difference was relatively small, with median values of 7.680 (absence of *H. pylori*) versus 7.570 (presence of *H. pylori*). Nevertheless, the results still suggest that this type of pathogen prefers a neutral or weakly acidic environment. A previous study reported that the suitable pH range for *H. pylori* is 5.5–7.5 [29]. Thus, the absence of *H. pylori* in the present study may be related to the pH of the water bodies.

The *Helicobacter* species found in the present study were mainly enterohepatic. Thus, their presence may be associated with total coliform count and *Escherichia coli*, which are indicators of fecal pollution [30]. By contrast, Table 4 suggests that the presence of *Helicobacter* species was not associated with total coliform count. Hence, the high positive rate in water and the high tolerance of this group of pathogens [31] may lead to statistical bias. This issue warrants clarification in future studies targeting relatively clean streams.

Wastewater can test positive for *Helicobacter* species at a rate of up to 84% [32], suggesting that these pathogens are inherently more prevalent in wastewater than in other environments. In the present study, most of the sampling sites that tested positive in this study were surrounded by livestock farms, confirming that the positive rate of this group of pathogens was higher in the surrounding streams because of the absence of proper wastewater treatment and sewage pipelines.

The river pollution index (*p* = 0.087) also proved to be a critical factor influencing the presence or absence of *Helicobacter* species. *Helicobacter* species cannot be completely eradicated by conventional cleaning and disinfection techniques [31]. Thus, the detection of this pathogen at TKC2, which is a drinking water source, would pose a significant risk to public health.

Only one strain of gastric *Helicobacter*, *H. turcicus*, was detected in this study, and *H. pylori* was not detected. This may be due to the fact that animal feces are the main source of pollution in both sampled watersheds and that the enterohepatic *Helicobacter* group is predominant in animal feces, whereas *H. pylori* or gastric *Helicobacter* is very rare [33]. In addition, *Helicobacter* species possibly exist as a single species in water bodies because of competition among organisms of the same species in the environment, as observed in *Listeria monocytogenes/innocua* [34], and the influence of the pH of the water body and the environment. However, these hypotheses need to be verified in subsequent studies.

All the remaining *Helicobacter* species, including *H. anseris*, *H. valdiviensis*, *H. felis*, *H. canadensis*, *H. pullorum*, and *H. canis*, were enterohepatic. These strains have gained considerable attention in the current society primarily because of their association with gastrointestinal diseases in humans [35]. In the present study, the most abundant *Helicobacter* strain identified in the Yanshui and Donggang rivers was *H. kayseriensis*, which is a novel strain found in the feces of wild birds [36]. *H. valdiviensis*, which was first detected in wild bird droppings, is a potential pathogen of intestinal infections in humans [37]. *H. canadensis* and *H. pullorum* have been found in human droppings [36,38], and *H. felis* has been detected mainly in feline animals [39]. However, phylogenetic tree analysis showed that the *H. winghamensis*, *H. canadensis*, and *H. valdiviensis* were classified into the same cluster (Cluster 11), implying that these *Helicobacter* species share a similar relationship. A previous study in Canada reported the presence of *H. winghamensis* in the feces of patients with inflammatory gastrointestinal disease and has a similar morphology to *Campylobacter* [5]. *H. winghamensis* has also been detected in sheep and pet dogs [2,40]. *H. winghamensis* lineage and *H. kayseriensis* lineage represented the two most common NHPH group. In the present study, Table 1, Table 3 show that almost all of the detected *H. kayseriensis* strains were located in the Donggang River, which belongs to a developed area of livestock farming. Interestingly, the *H. kayseriensis* detected in the Yanshui River was not found in the same area as *H. winghamensis* linage (Cluster 11) but was distributed in the vicinity of the farms, suggesting that this type of pathogen could be associated with animal husbandry. However, whether zoonotic transmission is a concern requires further research.

In Taiwan, the proportion of enterohepatic *Helicobacter* species in the feces of pet dogs is as high as 69.4%, whereas *H. pylori* accounts for approximately 26.4%, indicating that enterohepatic *Helicobacter* species remains predominant in the feces of most pet dogs, with *H. canis* being the most common [2]. This finding suggests a potential threat for animal–human and water–human transmission. YSC3-Sp in the present study is a *H. anseris* strain widely distributed in various wild birds and geese [41]. *H. trogontum* and *H. anseris* have been associated with diarrhea and bacteremia in humans [41,42], whereas *H. trogontum* is mainly isolated from mice [42]. TKC6-Sp and YSC3-W in the present study are *H. trogontum* strains mainly distributed in the middle and upper reaches of the Yanshui and Donggang rivers, both of which are affected by agricultural and livestock pollution. Samples TKC1-Sp, TKC5-A, YSC2-Sp, YSC5-S, and YSC2-S from the Yanshui and Donggang rivers were classified in the same cluster as *H. rodentium*, and TKC5-A and YSC2-S were located in the periphery of aquaculture/drinking water sources, which raised the suspicion of food- or water-borne transmission. *H. rodentium*, a strain detected in rats, was found in the watersheds of the Yanshui and Donggang rivers. A previous study reported its presence in the human liver, possibly contributing to chronic hepatitis development [43]. Results of the present study indicated that water bodies with specific environmental conditions were associated with the presence of *Helicobacter* species, and *Helicobacter* species from wild birds were found throughout the streams. Therefore, special attention should be paid to *Helicobacter* contamination if these streams were to be used as a source of drinking water to avoid pathogen transmission.

In the present study, eight amplicons were identified using *Helicobacter* 16 s rRNA PCR sequencing, and the sequences were found to be related to those of uncultured bacteria (AY592572.1) and *Q. citrea* (CP098494.1) from the NCBI database. Among them, TKC6-S, TKC5-S, YSC1-A, YSC2-A, and TKC4-W were more similar to an uncultured bacterium (AY592572.1) that was shown to be an unknown bacterium present in the volcanic substrate [20]. The maximum identities of TKC4-A, YSC2-W, and TKC1-W with *Q. citrea* (CP098494.1) ranged from 89% to 91%, and these strains were suggested to be a type of *Helicobacter* species present in the mud sediment [21]. However, none of them showed a high degree of similarity. Therefore, these strains may be identified with other *Helicobacter* species or bacteria, which requires further research and analysis for identification. These amplicons were mainly distributed in the middle and lower reaches of the Yanshui and Donggang rivers, which are densely populated settlements and areas with some farmland where large amounts of domestic livestock wastewater may be generated and possibly associated with wastewater contamination.

The major types of pollution in the Yanshui and Donggang rivers include agricultural, livestock, aquaculture, and domestic sewage pollution. The sampling points for agricultural and livestock pollution were TKC6, TKC5, TKC4, TKC3, YSC5, YSC4, YSC4, YSC5, YSC4, YSC5, YSC5, YSC4, and YSC5. Livestock pollution was reported in TKC6, TKC5, TKC4, TKC3, YSC5, YSC4, YSC3, and YSC2, and *H. kayseriensis* (6/16, 37.5%) was the most prevalent *Helicobacter* species. *H. kayseriensis* is often found in animal feces, suggesting that contamination at these sampling sites could be mainly related to animal husbandry [36]. It also implies that sources of contamination could be identified from the types of *Helicobacter* species. For instance, some of the types related to animal husbandry could be used as a reference for contamination of livestock wastewater. The sampling site related to aquaculture pollution was YSC1, and the main amplicons were compared to *H. kayseriensis* and uncultured bacteria. Positive samples that were more similar to uncultured bacteria need further investigations to determine whether they are novel *Helicobacter* strains. *H. kayseriensis* is mainly associated with livestock feces, and its presence in potential sites of aquaculture contamination suggests that the species is widely dispersed in streams with possible aquaculture contamination, given that the downstream outlets of the watershed are an important area for oysters and cultured fish. Some studies have detected *Helicobacter* species in fish, with a prevalence as high as 61.1% [44]. Thus, special attention must be paid to the threat of zoonotic infections caused by *Helicobacter* species*.*

In the present study, TKC1, TKC2, and YSC1 were likely to be affected by domestic sewage pollution, and the amplicons among the three sampling sites were dominated by *H. kayseriensis* (50%, 4/8), followed by *H. rodentium* (12.5%). Although some populated areas in these regions have well-established domestic sewage systems (data from the Construction and Planning Agency, Ministry of the Interior), most of them still lack these systems. Therefore, wastewater management is crucial to control the *Helicobacter* contamination of natural water bodies.

Part of Donggang River has been used as a water supply stream in Pingtung City, and its intake point is located at the Gangxi Pumping Station, which is also one of the sampling sites in this study (TKC2). The positivity rate of *Helicobacter* species in TKC2 reached 75%, and these species were not detected only in winter. The *Helicobacter* species detected after comparison were *H. kayseriensis* (NR_181912.1) and *Helicobacter* sp. (KJ081208.1), which were detected in magpie and *Cordylus warreni*, respectively [36,45].

*Helicobacter* spp. contamination in tap water could cause infections in humans. The Yanshui River is a future source of tap water, and downstream area is currently an important aquaculture area in Taiwan. This work showed the distribution of the zoonotic pathogen, *Helicobacter* spp., in two important and widely utilized streams. From the perspective of One Health, the interconnection between environmental health, animal health, and human health is crucial, particularly concerning zoonotic infectious diseases. Hence, continuous monitoring of pathogens and improvement of wastewater treatment systems are warranted to minimize the potential threat of *Helicobacter* species infection to humans.

## 5 Conclusions

1. *H. kayseriensis* was the most prevalent *Helicobacter* species (10/35, 28.57%) in the Yanshui and Donggang rivers, making it an important indicator of *Helicobacter* contamination in the area.
2. The main *Helicobacter* species identified in both rivers were enterohepatic. Some of the amplicons need to be identified in subsequent isolation and culture experiments.
3. *Helicobacter* species were detected at the tap water collection point in Donggang River TKC2, with *H. kayseriensis*, usually derived from animal species, as the most predominant species. Therefore, animal feces could be an important indicator of *Helicobacter* contamination in river water.
4. Based on the types and sources of contamination, livestock wastewater was estimated to be the main source of water contamination by *Helicobacter* species.

## Consent for publication

Not applicable.

## Funding

This work was supported by the E10.13039/501100004738-Da Hospital (grant number EDAHS111008, EDAHT112008 and EDPJ111040), I-Shou University (grant number ISU113-IND011-009 & ISU-113-01-12A) and National Science and Technology Council, Taiwan (grant number 111–2314-B-650 -001 -MY2 & 110-2314-B-650 -011 -MY2). The funding bodies played no role in the design of the study and collection, analysis, interpretation of data, and in writing the manuscript.

## Declaration of competing interest

The authors declare that they have no known competing financial interests or personal relationships that could have appeared to influence the work reported in this paper.

## Data Availability

Data will be made available on request.

## Appendix A Supplementary data

Supplementary material Image 4
