# Supplementary material for: Molecular surveillance of Helicobacter species with high prevalence from two streams with various wastewater pollution in Taiwan
Source: One Health. 2024 May 16;18:100757. doi: 10.1016/j.onehlt.2024.100757 (PMC11128502; doi:10.1016/j.onehlt.2024.100757)
Supplement: Supplementary file 1 — Supplementary material [file mmc1.docx]

Table S1

Sampling information in the YanShui river and Donggang river.

| Sample | Latitude and Longitude | Wastewater source | Sample number | | |
| --- | --- | --- | --- | --- | --- |
| YSC1 | 23°1'11.183"，120°11'32.885" | Domestic sewage pollution、Aquatic pollution | | 4 |  |
| YSC2 | 23°1'29.968"，120°12'55.966" | Agricultural pollution、Livestock pollution | | 3 |  |
| YSC3 | 23°3'32.702"，120°16'39.997" | Agricultural pollution、Livestock pollution | | 4 |  |
| YSC4 | 23°4'20.280"，120°21'29.700" | Agricultural pollution、Livestock pollution | | 4 |  |
| YSC5 | 23°03'33.179"，120°21'47.540" | Agricultural pollution、Livestock pollution | | 4 |  |
| TCK1 | 22°28'39.781"，120°27'34.686" | Domestic sewage pollution | | 4 |  |
| TCK2 | 22°30'16.283"，120°27'43.225" | Domestic sewage pollution | | 4 |  |
| TCK3 | 22°32'31.916"，120°30'14.990" | Agricultural pollution、Livestock pollution | | 4 |  |
| TCK4 | 22°33'49.637"，120°32'22.779" | Agricultural pollution、Livestock pollution | | 4 |  |
| TCK5 | 22°36'10.566"，120°34'58.627" | Agricultural pollution、Livestock pollution | | 4 |  |
| TCK6 | 22°36'49"，120°35'19.3" | Agricultural pollution、Livestock pollution | | 4 |  |
